# The hidden cost of patient feedback: analysis of a decade of NHS expenditure on orthopaedic PROMs

**DOI:** 10.1186/s41687-026-01052-x

**Published:** 2026-04-27

**Authors:** Patrick Cook, Mohammad Alhashash, Christopher Wakeling

**Affiliations:** Trauma & Orthopaedics, University Hospitals Sussex NHS Foundation Trust, Brighton, UK

**Keywords:** PROMs, Arthroplasty, Freedom of information

## Abstract

**Background:**

Patient-Reported Outcome Measures (PROMs) have become integral to assessing care quality, shifting emphasis from surgeon-centric metrics to patient-centred outcomes. Within the National Health Service (NHS) of the United Kingdom, routine PROMs collection for hip and knee arthroplasty has been mandated since 2009, but rising costs, falling completion rates, and operational inefficiencies raise concerns over sustainability. With completion rates for hip arthroplasty falling from 67% in 2014 to 27.6% in 2024, the financial cost for PROMs must be assessed. This study aimed to quantify the financial burden of PROMs collection across NHS orthopaedic services in England over the past decade and evaluate implications for policy, resource allocation, and digital transformation planning.

**Methods:**

A retrospective cost analysis was conducted of all NHS trusts in England listed on the National Joint Registry (NJR), excluding private providers. Freedom of Information (FOI) requests sought annual PROMs-related expenditure from 2015 to 2025, itemising internal staffing and third-party contractor costs. Trusts lacking complete cost breakdowns or exceeding FOI statutory work limits were excluded from total cost calculations but contributed to component-level analyses where possible. National costs were estimated using two extrapolation approaches (per-trust and per-procedure) and presented alongside sensitivity analyses. All trend analyses were performed using costs expressed in 2024 prices after adjustment for inflation with the Consumer Price Index (CPI); nominal costs are also reported to reflect actual NHS cash expenditure.

**Results:**

Thirty-four trusts provided full data, representing 40.7% (41,293/101,525) of NHS arthroplasty procedures in England. After adjusting for CPI inflation, annual observed PROMs expenditure among these trusts rose from £1.144 million in 2015 to £1.39 million in 2024 with a real-terms increase of 21%, indicating that the majority of the nominal rise reflects inflationary pressures, though real cost growth persists. Modelled NHS-wide cumulative expenditure over ten years was estimated at between £29.1 million and £39.1 million, depending on extrapolation method. A total of 42 trusts provided third-party contracting costs. Contractor costs increased from a mean of £15,000 per trust in 2015 to £19,921 in 2024. Average trust internal staffing costs remained static averaging £32,068 in 2015 to £33,200 in 2024.

**Conclusion:**

PROMs are essential for patient-centred orthopaedic care but are associated with escalating costs, falling completion rates, and fragmented administration. Centralised procurement, NHS digital integration, dedicated staffing, and hybrid collection models are recommended to improve efficiency, data quality, and long-term sustainability. National expenditure estimates should be interpreted cautiously given the 32% response rate and reliance on modelling assumptions.

## Background

The evolution of assessing orthopaedic care has undergone a seismic shift from surgeon-centric metrics to patient-centred outcomes over the past two decades. Where once success was measured purely through implant survival rates and radiographic appearances, the emergence of Patient-Reported Outcome Measures (PROMs) has fundamentally redefined quality assessment. Arthroplasty is performed to improve a patient’s pain, function and quality of life thus measurement of these factors is essential when assessing surgical success. This transformation reflects broader healthcare trends toward personalised medicine and value-based care models that prioritise the patient experience alongside clinical effectiveness.

Since the introduction of routine PROMs collection for hip and knee arthroplasty within the NHS in 2009, PROMs have become an integral component of orthopaedic practice, informing both clinical decision-making and health policy. However, the implementation of PROMs has not been without challenge. Early evaluations revealed significant administrative difficulties, with up to 14% of PROMs affected by errors and 23% containing incomplete data [1]. Literature including the EMPROV study, have highlighted issues such as respondent fatigue and declining completion rates [2]. When assessing the completed questionnaires, defined as a pre and post procedure PROM linked to a healthcare episode, completion rates have fallen from 67% in 2014 to 27% in 2023 [3]. Furthermore, reliance on unsupervised paper-based PROMs has been associated with missing, illegible, or unreliable data [4], raising concerns about data integrity and the overall applicability of PROMs in practice.

As healthcare leaders consider expanding PROMs collection to encompass a wider range of procedures and medical devices, orthopaedic clinicians, drawing on over a decade of experience, are uniquely positioned to inform best practice. The increasing volume of arthroplasty procedures, estimated at nearly 40% for both hip and knee procedures compared to 2018 volumes, driven by an ageing population, is expected to further amplify the logistical and financial demands of PROMs collection [5]. Whilst there is growing interest in the digitalisation of PROMs to improve efficiency and reduce costs, significant barriers remain. Notably, digital illiteracy affects approximately 50% of individuals over the age of 70 [6], necessitating careful consideration of equity and accessibility in any transition to digital platforms. England is not unique in the national collection of PROMs. The Swedish Hip Quality Register (SHPR) and Swedish Knee Arthroplasty Register (SKAR) have routinely collected this data since 2002 but differ in that collection was integrated into these registers while in England it was launched independently from the National Joint Registry (NJR). There has been interest in the best methods to collect PROMs data; cheaper automated digital solutions, hybrid systems whereby if digital collection is not successful the patient is telephoned and finally traditional written surveys. It is unclear how the method influences completion rates; one study demonstrating that the minimal effort automated digital system had a completion rate of 53% at one year compared to the hybrid system’s 83% completion rate. However, an analysis of the Swedish Joint Registers shows significant variation in completion rates from 32% to 88% despite using the same collection method [7]. The Swedish data would therefore suggest that patient pathways are a greater influence on completion rates than the specific method of collecting PROMs.

PROMs collection practices vary considerably across NHS trusts in England, with some providers managing the process internally, others outsourcing to accredited third-party suppliers, and a minority employing a hybrid approach. Only 21% of trusts currently have a dedicated PROMs manager [2], and the regulatory landscape has shifted to allow for increased involvement of private suppliers. This fragmentation complicates efforts to accurately assess the true costs of PROMs collection, with previous estimates suggesting an annual expenditure of approximately £800,000 a decade ago [8]. In the absence of centralised cost reporting, a comprehensive understanding of current PROMs-related costs within orthopaedics remains elusive.

Given the NHS’s commitment to a £2.1 billion digital transformation during 2025, there is a need to assess the economic implications and implementation challenges associated with PROM modernisation. This study aims to address this knowledge gap by providing a detailed analysis of PROMs collection costs across NHS orthopaedic service providers in England, thereby informing policy development and resource allocation as the healthcare system moves towards greater digital integration.

This study was previously presented as an oral presentation at the 2025 British Orthopaedic Association Meeting on September 16, 2025.

## Materials and methods

This study employed a retrospective cost analysis to evaluate the financial burden associated with the collection of PROMs across NHS trusts in England. All NHS trusts listed on the National Joint Registry (NJR) were identified as the study population (*n* = 107). All private providers were excluded from the study. Data collection was conducted via Freedom of Information (FOI) requests, which were electronically distributed to each trust. The FOI requests specifically sought detailed information on the total costs incurred for PROMs collection over the preceding ten-year period (2015–2025), with an itemised breakdown distinguishing between internal staffing costs and expenditures associated with third-party contractors.

Trusts were included in the analysis if they provided data on both internal and third-party PROMs collection Costs for the full ten-year period. Trusts were excluded if they were unable to provide a cost breakdown, if the information was deemed commercially sensitive, or if retrieval of the data would exceed the statutory maximum labour time (eighteen hours) for FOI requests. If a trust could provide the third-party costs only, the trust would be excluded from the total cost analysis but included in the calculation of mean third-party costs. In cases where trusts did not respond to the FOI request or reported that no records were available, these trusts were also excluded.

### Cost definitions

FOI requests asked trusts to report two categories of PROMs-related expenditure:

- **Internal staffing costs**: salary and on-costs for staff whose time is dedicated, in whole or in part, to PROMs administration, data entry, patient contact, and quality assurance. This may include dedicated PROMs co-ordinators, administrative staff, and (in a minority of trusts) clinical staff with protected PROMs time.
- **Third-party contractor costs**: amounts invoiced by accredited external PROMs suppliers for data collection, processing, and reporting services.

#### Excluded costs

The FOI request did not capture indirect costs such as unfunded clinician time spent administering PROMs during consultations, IT infrastructure or software licensing not billed separately for PROMs, printing and postage absorbed into general departmental budgets, or the opportunity cost of patient or staff time. Because there is no nationally standardised costing framework for PROMs, individual trusts may have classified borderline costs (e.g., shared administrative roles) differently. Reported costs should therefore be regarded as approximations of direct PROMs expenditure rather than comprehensive economic costs. This limitation is discussed further in the Limitations section.

### Procedural volume and completion rate data

Procedural volume was captured from the NJR website for each trust. PROM completion rates for each trust were obtained from the NHS Digital website, with the information publicly available.

### Non-response analysis

To assess the potential for non-response bias, we compared responding trusts (*n* = 34) with non-responding or excluded trusts (*n* = 73) on available characteristics: mean annual procedural volume (from the NJR) and geographic region. The comparisons are descriptive and do not constitute a formal bias adjustment but provide context for interpreting the representativeness of the included sample.

### Inflation adjustment

All costs are reported in both nominal (as-reported) terms and real terms adjusted to 2024 prices. For the primary analysis, we used the Consumer Price Index (CPI). CPI-adjusted figures were used for all temporal trend analyses, figures, and interpretive conclusions, while nominal figures were retained to show the actual cash outlay borne by NHS organisations. Over the study period 2015–2024, the cumulative inflation factor was approximately 1.33 (i.e., £1 in 2015 is equivalent to approximately £1.33 in 2024).

### Statistical analysis

Linear regression analysis was used to describe the trend in mean CPI-adjusted trust-level PROMs cost over time (2015–2024) expressed in 2024 prices. This analysis is descriptive and characterises the direction and magnitude of cost change; it does not model causation or directly quantify sustainability. To directly assess the value-for-money dimension, we calculated the cost per completed PROM for each year by dividing total observed expenditure by the number of completed PROMs (defined as linked pre- and post-operative questionnaire pairs, obtained from NHS Digital). This metric captures the interaction between rising costs and falling completion rates. All analyses were performed in IBM SPSS for Macintosh version 31. A p-value of less than 0.05 was considered statistically significant. Where regression coefficients are reported, 95% confidence intervals (CIs) are provided.

### National extrapolation

National expenditure was estimated using two approaches to test the sensitivity of the extrapolation:

1. Per-trust extrapolation: The mean cost per responding trust was multiplied by the total number of eligible trusts.
2. Per-procedure extrapolation: The mean cost per arthroplasty procedure among responding trusts was multiplied by total national procedural volume from the NJR.

Both approaches assume that non-responding trusts have similar cost profiles to responding trusts. The plausibility of this assumption is discussed in the limitations sub-section, and both estimates are presented as a range to convey extrapolation uncertainty. These figures are explicitly labelled as modelled estimates throughout.

### Ethical approval

Ethical approval was not required for this study, as all data were obtained from publicly accessible sources and did not involve patient-level information.

## Results

A total of 107 trusts were sent a FOI request. Thirty-four NHS trusts (34/107, 32%), provided sufficient data for inclusion with an average annual procedural volume of 41,293 cases. Consequently, the dataset encompassed 41% (41,293/101,525) of total arthroplasty case volume recorded in NHS England hospitals.

A further nine trusts (9/107, 8.4%) could provide third-party contractor costs only with the internal staffing costs unknown.

Three trusts (3/107, 2.8%) withheld cost data citing commercial sensitivity and 25 trusts (25/107, 24%) failed to respond to the Freedom of Information request despite initial acknowledgment. Finally, 36 trusts (36/107, 34%) did not hold costings data or collection of this data would exceed the 18 h of labour stipulated by the FOI Act.

### Non-response comparison

Responding trusts (*n* = 34) had a mean annual procedural volume of 1,215 hip and knee arthroplasties, compared with an estimated mean of 826 among non-responding trusts, suggesting that larger trusts were more likely to provide complete data. The geographic distribution of responding trusts spanned all NHS regions in England, though with slight over-representation of trusts in large metropolitan areas. These differences mean that the included sample may not be fully representative of all NHS trusts, and the results should be interpreted with this in mind.

### Total observed expenditure

**Fig. 1:**
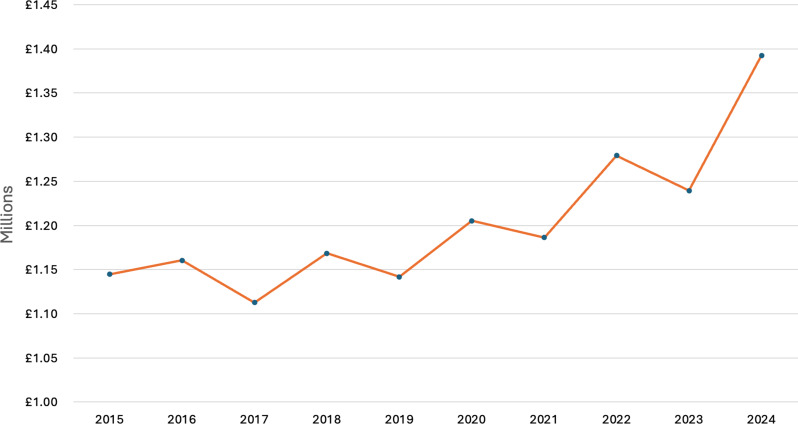
Total annual PROMs expenditure reported by 34 trusts (CPI adjusted values, £)

Annual PROMs-related expenditure among trusts that could provide complete data increased from £1.1 million in 2015 to £1.39 million in 2024 after CPI adjustment, indicating an approximate 21% real-terms increase over the study period. Thus, while a substantial proportion of the nominal rise from reflects inflation, real growth in PROMs expenditure remained evident and this is visible in Fig. 1.

### Mean cost per trust

**Fig. 2:**
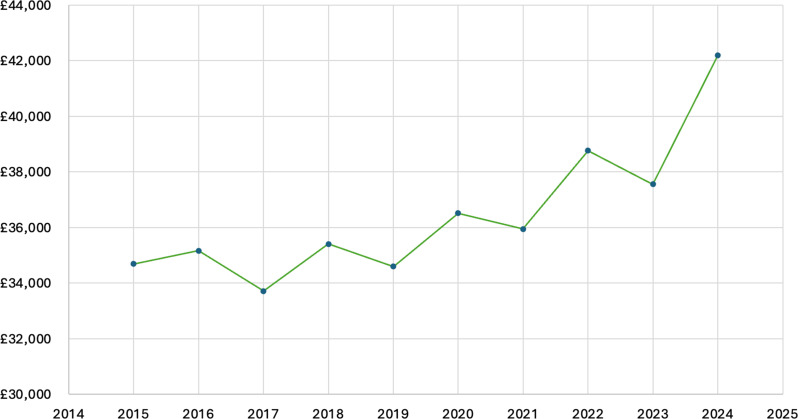
Mean annual PROMs cost per trust (CPI adjusted, £), 2015–2024. Data from 34 trusts with complete cost data

Correspondingly, the mean cost to each NHS trust using CPI adjusted values rose from £34,692 in 2015 to £42,195 in 2024 and this is shown in Fig. 2.

### Third-party contractor costs

A total of 43 trusts provided third-party contracting costs. After CPI adjustment to 2024 prices, this represented an increase from an average of £15,000 to £19,900, corresponding to an approximate 33% real-terms increase.

### Internal staffing costs

Internal costs were provided by 34 trusts. After CPI adjustment, this represented an increase from approximately £31,500 to £33,200, corresponding to an approximate 5% real-terms increase, consistent with inflation accounting for most of the rise.

### Modelled national estimates

The following figures are modelled estimates, not observed data. In nominal terms, cumulative NHS expenditure over the ten-year period was estimated at £24.6 million to £32.7 million, depending on extrapolation method. For interpretation of time trends, CPI-adjusted estimates expressed in 2024 prices were used in the main analysis. They are derived by extrapolating from the responding trusts and should be interpreted with the caveats described in the Limitations section.

### Approach: Per-trust extrapolation

Multiplying the mean cost per responding trust by 107 eligible trusts yields an estimated total cumulative NHS expenditure of £39.0 million over the ten-year period, with annual costs rising from an estimated £3.64 million in 2015 to £4.5 million in 2024 CPI adjusted values.

### Approach: Per-procedure extrapolation

**Fig. 3:**
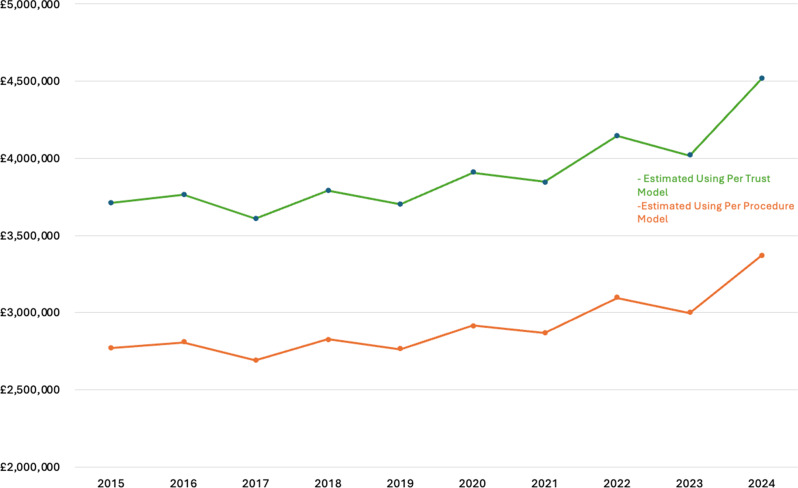
Estimated total annual NHS PROMs expenditure in England, 2015–2024 (modelled, CPI adjusted values £). (These are extrapolated estimates based on per-trust and per-procedure methods applied to data from 34 responding trusts. These figures were not directly observed.)

Dividing total observed expenditure by total procedural volume among responding trusts yields a mean cost per procedure, which is then multiplied by national NJR volume. This yields an estimated total cumulative NHS expenditure of £29.1 million over the ten-year period, with annual costs rising from an estimated £2.73 million in 2015 adjusting to inflation to £3.4 million in 2024 (Fig. 3).

### Sensitivity range

Combining both approaches, the estimated cumulative national expenditure on orthopaedic PROMs collection over 2015–2024 lies in the range of £29.1 million to £39.0 million. This range does not constitute a formal confidence interval but reflects the sensitivity of the estimate to the extrapolation method employed.

## Discussion

The integration of PROMs into orthopaedic practice marks a significant paradigm shift toward patient-centred care, aligning with broader healthcare trends emphasizing value-based outcomes and personalized medicine. However, the findings of this decade-long cost analysis reveal substantial systemic inefficiencies and escalating financial burdens that challenge the sustainability PROM programmes within NHS England. Among the 34 trusts providing complete data, total annual PROMs expenditure rose by 21% after adjustment for inflation over the study period and underscores a concerning trend of rising costs despite stagnant or declining response rates.

Our national cost estimates are extrapolated from a subset of trusts and may not be representative of the full NHS landscape. Responding trusts were, on average, larger than non-responding trusts, which could bias the per-trust extrapolation upward or downward depending on economies of scale. The sensitivity range of £29.1 million to £39.0 million over ten years reflects this uncertainty. While staffing costs have gone up steadily, the surge in contractor expenses has played the dominant role in overall cost escalation, raising concerns about long-term sustainability and efficiency of current practices.

The marked rise in contractor costs suggests that reliance on external providers has become an increasingly significant driver of PROM-related expenditure. The disproportionately large increase in contractor costs compared to staffing likely reflects a sector-wide shift away from internally managed PROMs to outsourced solutions. Formal benchmarking of per-unit contractor tariffs was not possible due to the constraints of FOI reporting and commercial confidentiality. However, informal qualitative responses from several trusts indicate that many have moved from per-unit pricing models to block payment contracts with their PROMs providers. Given that most trusts are also now funded through block payment mechanisms rather than activity-based best practice tariffs, the observed increase in contractor costs is unlikely to be explained solely by an expansion in the scope of outsourced activity.

Internal staffing costs showed a more predictable and stable rise over time. The rise in PROMs costs was driven predominantly by third-party contractor expenditure rather than internal staffing costs. Although contractor costs rose substantially in nominal terms, the more important analytical finding is that they also increased by approximately 40% in real terms, whereas staffing costs increased by only around 5% after inflation adjustment. This pattern suggests that outsourcing, rather than workforce expansion alone, has been the principal driver of real-terms cost escalation. A critical factor undermining investment incentives has been the shift from activity-based best practice tariff to block payment structures. When PROMs were first mandated in 2009, completion rates directly influenced reimbursement under tariff-based funding, incentivising trusts to maximise data collection quality and completion. However, with most NHS trusts now operating under fixed block contracts, this financial driver has largely disappeared, contributing to the observed decline in completion rates and institutional deprioritisation of PROM programmes. This policy shift inadvertently severed the link between PROM performance and resource allocation, effectively removing accountability mechanisms.

The pronounced rise in third-party costs raises important questions about the cost-effectiveness of external procurement strategies. If contractor charges continue to grow faster than in-house staffing budgets, resource allocation could become increasingly inefficient, threatening the sustainability of PROMs programmes. Given declining completion rates and fragmented collection practices, there is an urgent need for policy reform. Centralising procurement and digitalising PROMs workflows, combined with targeted investments in dedicated staff and hybrid collection models, offer potential to contain costs and drive improvements in both efficiency and data quality. A centralised procurement with digitisation of PROM collection presents a promising solution to reduce administrative burden and improve data integrity. the clinical utility of PROMs must also be critically appraised in the context of these rising costs. The literature consistently highlights respondent fatigue and data quality concerns, particularly with unsupervised paper-based collection methods [4].

Furthermore, the NJR annual report highlights the need to better integrate this data with PROM collection and centralised procurement may further aid this effort as has been done in other countries [9]. Pilot initiatives integrating PROMs with digital platforms such as the Surgical Devices and Implants Information System (SDIIS) could demonstrate potential efficiencies.

Nevertheless, demographic realities pose significant challenges; approximately 4.7 million adults over 65 lack basic digital literacy, necessitating hybrid collection strategies to maintain inclusivity and data completeness [10]. Alternatively, if surgeons & Healthcare Leaders accept lower completion rates from a purely digital system, with literature suggesting a rate of 53% at one year, costs could be reduced significantly [11]. The expansion of PROMs beyond hip and knee arthroplasty to encompass other surgical procedures and medical devices further complicates the landscape. While orthopaedics’ extensive experience positions the specialty to lead this expansion, it also raises concerns about escalating workload and the need for dedicated PROM management personnel. Currently, only 21% of trusts employ dedicated PROM managers, with most responsibilities absorbed into broader administrative or other staff roles, risking compromised data quality and staff burnout [3].

Beyond structural and funding reforms, targeted design of PROMs programmes could also reduce respondent burden and improve engagement. Computerised adaptive testing (CAT) has been shown to shorten questionnaires substantially by tailoring item selection to the individual patient, while maintaining measurement precision, and therefore offers a pragmatic route to decreasing completion time and fatigue [12]. Individualised feedback, such as presenting patients with graphical summaries of their scores over time or benchmarking against expected recovery trajectories, may further enhance perceived relevance and encourage sustained participation [13]. In England, integrating PROMs capture and feedback into the NHS App could streamline data collection, provide real-time feedback to patients, and align PROM completion with existing digital touchpoints in the patient pathway. Emerging applications of generative artificial intelligence may also support PROMs workflows by automating reminder scheduling, triaging free-text responses, and generating concise summaries for clinicians and service managers. While these technologies require careful governance, validation, and attention to equity, they represent promising tools to reduce administrative burden and help convert PROMs data into actionable insights at both patient and service levels.

### Limitations

This study has several limitations that should be considered when interpreting the findings. First, only 34 of 107 eligible trusts provided complete cost data, raising the possibility of selection and non-response bias and limiting the representativeness of our sample. When adjusting for arthroplasty volume, these trusts accounted for a large proportion and therefore the sample may over-represent larger organisation. Trusts without accessible cost records may have less structured PROMs programmes, potentially with different cost trajectories.

Second, FOI responses were not based on a standardised costing framework, and we could not fully verify which cost components each trust included; the estimates should therefore be interpreted as approximations.

Third, national expenditure figures are modelled rather than observed; they rely on the assumption that non-responding trusts have similar cost structures to responders, and are sensitive to whether extrapolation is performed per trust or per procedure. We present a range derived from per-trust and per-procedure extrapolation to illustrate this sensitivity, but this range does not constitute a formal confidence interval and does not capture all sources of uncertainty. However, our costing data does broadly correspond with the estimates made with a decade ago by NHS England, providing some external validation that the broad trends are consistent with the true picture [8]. The distinction between nominal and real-terms costs is important for policy interpretation. Nominal figures reflect the actual cash expenditure faced by NHS organisations and are therefore relevant to service planning and budget impact, whereas CPI-adjusted figures provide the appropriate basis for evaluating temporal trends and sustainability. On that basis, the present findings suggest that current PROMs collection models have become more expensive in real terms over time, strengthening the case for procurement reform, digital integration, and more efficient hybrid collection pathways.

Considering these findings, it is imperative to optimise PROMs collection frameworks, emphasised by the declining completion rate and, as this study now shows, rising costs. Centralised procurement and standardisation via NHS digital platforms could streamline processes and contain costs but must be accompanied by strategic investment in dedicated staffing and robust training. Furthermore, minimizing duplication of data collection and aligning PROMs with clinical workflows will enhance both efficiency and clinical relevance. Centralisation of PROMs collection would also enable sharing with clinical researchers to facilitate robust assessment in a variety of clinical studies and avoid duplication of collection - which would further risk responder fatigue. Finally, ongoing evaluation of PROM impact on patient outcomes and healthcare resource allocation is essential to justify continued investment.

## Conclusions

This comprehensive cost analysis reveals a concerning trajectory for PROMs collection in the United Kingdom; increasing expenditure coupled with declining completion rates. This cost escalation occurs against a backdrop of disappearing financial incentives following the shift from activity-based tariff to block payment structures, effectively severing accountability mechanisms that previously drove data quality. Current PROMs infrastructure is financially unsustainable, particularly given the disproportionate increase in contracting costs on a background of steady increases in internal staffing expenditure.

Despite these challenges, PROMs remain fundamental to patient centred orthopaedic care and value-based healthcare delivery. If linked successfully with the joint registries, implants could be evaluated for how they improve quality of life as opposed to just if they need revising. Integration with existing infrastructure such as the National Joint Registry and Surgical Devices and Implants Information System would eliminate redundancy and facilitate research accessibility. The solution lies not in abandoning PROMs but in fundamental structural reform. Centralised procurement through NHS Digital platforms offers an immediate opportunity to reduce administrative duplication and contractor dependency whilst improving data integrity.

With arthroplasty volumes projected to increase 40% and NHS Digital transformation receiving £2.1 billion investment, orthopaedic services are uniquely positioned to pioneer evidence-based PROM modernisation. The path forward requires coordinated policy reform prioritising centralisation, digitalisation, dedicated staffing, and hybrid methodologies—ensuring PROMs fulfil their promise of enhancing patient outcomes whilst achieving long-term financial sustainability.

## Data Availability

All data is available upon reasonable request.

## References

[1] NHS England. Provisional monthly patient reported outcome measures (PROMs) in England (2015) Accessed: 2025-08-01: https://digital.nhs.uk/data-and-information/publications/statistical/patient-reported-outcome-measures-proms/provisio

[2] Matthews A, Evans JP (2023) Evaluating the measures in patient-reported outcomes, values and experiences (EMPROVE study): a collaborative audit of PROMs practice in orthopaedic care in the United Kingdom. Ann R Coll Surg Engl 23:357–364 10.1308/rcsann.2022.0041 PMC10066647 35938506

[3] NHS England. Finalised patient reported outcome measures (PROMs) in England (2012) Accessed: 01-07-2025: https://digital.nhs.uk/data-and-information/publications/statistical/patient-reported-outcome-measures-proms/finalise

[4] Byrom B, Tiplady B (2016) Data Quality and Power in Clinical Trials: A Comparison of ePRO and Paper in a Randomized Trial. Bill Byrom, vol 1. Taylor Francis, London, p 29

[5] Matharu GS, Culliford DJ, Blom AW (2022) Projections for primary hip and knee replacement surgery up to the year 2060: an analysis based on data from The National Joint Registry for England, Wales, Northern Ireland and the Isle of Man. Ann R Coll Surg Engl 23:443–448 10.1308/rcsann.2021.0206 PMC9157920 34939832

[6] McCleary NJ, Wigler D, Berry D et al (2013) Feasibility of computer-based self-administered cancer-specific geriatric assessment in older patients with gastrointestinal malignancy. Oncologist 23:64–72. 10.1634/theoncologist.2012 10.1634/theoncologist.2012-0241 PMC3556258 23287880

[7] Coster M, Bremander A, Nilsdotter A (2023) Patient-reported outcome for 17,648 patients in 5 different Swedish orthopaedic quality registers before and 1 year after surgery: an observational study. Acta Orthop 94:1–7. 10.2340/17453674.2023.6577 36701121 10.2340/17453674.2023.6577 PMC9880767

[8] National patient reported outcome measures (PROMs) Programme Consultation (2016) Accessed: 01-07-2025: https://www.engage.england.nhs.uk/consultation/proms-programme/

[9] National joint registry annual report (2024). Accessed: 01-07-2025: https://www.njrcentre.org.uk/national-joint-registry-annual-report-2024/

[10] Offline and Overlooked (2024) Accessed: 01-07-2025: https://www.ageuk.org.uk/latest-press/articles/2024/more-than-1-in-3-over-65s-4.7-million-lack-the-basic-skills-to-us

[11] Pronk Y, Pilot P, Brinkman JM, van Heerwaarden RJ, van der Weegen W (2019) Response rate and costs for automated patient-reported outcomes collection alone compared to combined automated and manual collection. J Patient Rep Outcomes 5:31 10.1186/s41687-019-0121-6 PMC6545294 31155689

[12] Evans JP, Gibbons C, Toms AD, Valderas HM (2022 Jul) Use of computerised adaptive testing to reduce the number of items in patient-reported hip and knee outcome scores: an analysis of the NHS England National Patient-Reported Outcome Measures programme. BMJ Open [Internet], p 20 10.1136/bmjopen-2021-059415 PMC9315912 35858721

[13] van Muilekom MM, Luijten MAJ, van Oers HA, Terwee CB, van Litsenburg RRL, Roorda LD et al (2021) From statistics to clinics: the visual feedback of PROMIS CATs. J Patient Rep Outcomes [Internet]. Dec 1 10.1186/s41687-021-00324-y PMC8272760 34245390

